# Using a population-based approach to prevent hepatocellular cancer in New South Wales, Australia: effects on health services utilisation

**DOI:** 10.1186/1472-6963-10-215

**Published:** 2010-07-21

**Authors:** Monica C Robotin, Melanie Q Kansil, Jacob George, Kirsten Howard, Steven Tipper, Miriam Levy, Nghi Phung, Andrew G Penman

**Affiliations:** 1 School of Public Health, University of Sydney NSW, Sydney, Australia; 2 Cancer Council NSW, Woolloomooloo NSW, Australia; 3 Centaurus Partners, Kensington NSW, Australia; 4 Storr Liver Unit, Millennium Institute, Westmead NSW, Australia; 5 School of Medicine, University of Sydney NSW, Sydney, Australia; 6 Department of Gastroenterology, Liverpool Hospital, Liverpool NSW, Australia; 7 University of New South Wales, Sydney NSW, Australia; 8 Departments of Gastroenterology and Addiction Medicine, Westmead Hospital, Westmead NSW, Australia

## Abstract

**Background:**

Australians born in countries where hepatitis B infection is endemic are 6-12 times more likely to develop hepatocellular cancer (HCC) than Australian-born individuals. However, a program of screening, surveillance and treatment of chronic hepatitis B (CHB) in high risk populations could significantly reduce disease progression and death related to end-stage liver disease and HCC. Consequently we are implementing the *B Positive *pilot project, aiming to optimise the management of CHB in at-risk populations in south-west Sydney. Program participants receive routine care, enhanced disease surveillance or specialist referral, according to their stage of CHB infection, level of viral load and extent of liver injury. In this paper we examine the program's potential impact on health services utilisation in the study area.

**Methods:**

Estimated numbers of CHB infections were derived from Australian Bureau of Statistics data and applying estimates of HBV prevalence rates from migrants' countries of birth. These figures were entered into a Markov model of disease progression, constructing a hypothetical cohort of Asian-born adults with CHB infection. We calculated the number of participants in different CHB disease states and estimated the numbers of GP and specialist consultations and liver ultrasound examinations the cohort would require annually over the life of the program.

**Results:**

Assuming a 25% participation rate among the 5,800 local residents estimated to have chronic hepatitis B infection, approximately 750 people would require routine follow up, 260 enhanced disease surveillance and 210 specialist care during the first year after recruitment is completed. This translates into 5 additional appointments per year for each local GP, 25 for each specialist and 420 additional liver ultrasound examinations.

**Conclusions:**

While the program will not greatly affect the volume of local GP consultations, it will lead to a significant increase in demand for specialist services. New models of CHB care may be required to aid program implementation and up scaling the program will need to factor in additional demands on health care utilisation in areas of high hepatitis B sero-prevalence.

## Background

Although hepatocellular cancer (HCC) remains relatively uncommon in Australia, its incidence has increased approximately five-fold since 1972; based upon current trends, a three-fold increase in the number of new cases is being envisaged by 2020 [1]. Primary liver cancer rates are highest in south-west Sydney, where incidence rates (7.7 per 100,000 persons) are significantly higher than the State average (5.2 per 100,000 persons) [1].

People with chronic viral hepatitis are at a significantly greater risk (20 to 200-fold) of developing HCC than those not infected [2,3] and worldwide, chronic infection with the hepatitis B virus (HBV) is responsible for approximately 50-55% of all liver cancers [4]. Approximately 90% of people infected in infancy develop chronic hepatitis B (CHB) infection, with most remaining asymptomatic until in late adulthood. Consequently most HCC diagnoses occur late, when treatment options are limited and survival rates are low [5].

People born in China and Vietnam represent a small proportion (approximately 5%) of the total Australian population, but carry a disproportionate burden of CHB-related disease, as > 50% of the total number of CHB cases in Australia are diagnosed in migrants born in one of these countries [6].

Furthermore, nearly 90% of hepatitis-B related HCC cases in NSW occur in people born overseas, and approximately 70% affect Australians born in countries of high hepatitis B prevalence [7]. Men born in Vietnam, Hong Kong, Macau, Korea, Indonesia and China and women born in Vietnam and China are 6-12 times more likely to develop HCC than non-Indigenous, Australian-born individuals [8].

So far only one large randomized controlled trial has reported a significant reduction in mortality (by 37%) in people with CHB followed up with biannual HCC screening [9] and in the absence of consensus on whether cancer screening reduces mortality [10], population-based screening for HCC is not practiced in Australia. Currently HCC diagnoses commonly occur late: a retrospective case series of liver cancers diagnosed in Western Sydney found that only a small proportion (< 20%) were detected by HCC surveillance, with most cases detected at an advanced stage [5].

Although recent evidence suggests that suppressing viral replication in CHB can delay the development of cirrhosis and may prevent liver cancer [11-13], current uptake of antiviral treatment in Australia remains low, with approximately 2% of people with CHB receiving antiviral therapy [14].

The geographic and ethnic clustering of CHB and HCC in Sydney's south-west provided an opportunity to devise a targeted public health intervention addressing the burden of disease associated with CHB in the area. Cancer Council NSW is piloting the *B Positive *program in this region, aiming to promote the early diagnosis and to optimize the management for CHB in primary care settings [15]. The program was developed with community consultation and input from general practitioners (GPs), medical specialists and researchers and was approved by the relevant Human Research Ethics Committees. The program is based in the five local government areas (LGAs) with the highest burden of hepatitis-B related HCC in NSW. In this paper we examine the potential impact of the *B Positive *program on health services utilisation in the study area.

## Methods

The B Positive project targets South East Asian residents from the Vietnamese and Chinese communities in south-west Sydney, but is inclusive of all individuals who meet eligibility criteria. Eligibility criteria include confirmed chronic hepatitis B, age ≥ 35 years and attending a general practice in the target local government areas (LGA).

**Figure 1:**
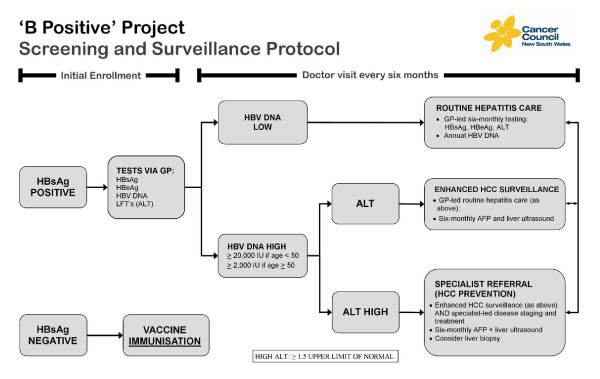
***B Positive *program screening and treatment algorithm**.

GPs conduct initial hepatitis B (HBV) testing and people with confirmed CHB are offered enrolment in the program. Participants are stratified into three risk categories, based upon their hepatitis B DNA (HBV DNA) and alanine amino transferase (ALT) levels (see Figure 1).

Low risk patients are characterised by low viral loads (defined as < 20,000 IU/ml for ages < 50 years and < 2,000 IU/ml for ages ≥ 50 years) and "normal" ALT levels - i.e. ALT levels < 1.5 times the upper limit of normal (ULN). They are enrolled in a program of *routine CHB surveillance*, consisting of six-monthly GP follow up, comprising a clinical examination and a review of blood test results for hepatitis B surface antigen (HBsAg), hepatitis B e antigen (HBeAg), viral load (VL) and ALT levels.

Intermediate risk patients have elevated HBV viral loads (≥ 20,000 IU for people aged under 50 years and ≥ 2,000 IU/ml for those age ≥ 50 years) and normal ALT levels. They are enrolled in a six-monthly *enhanced HCC surveillance program *with follow-up provided by their GP. Follow up routines are similar to the routine CHB surveillance, with additional biannual liver ultrasound (US) examinations and alpha fetoprotein (AFP) measurements.

High risk patients are characterised by elevated HBV viral loads and elevated ALT levels (both defined above). These patients are referred for specialist care, which includes CHB monitoring, HCC surveillance, as well as liver biopsy and pharmacologic treatment of CHB, as deemed necessary [15].

Viral load levels used for risk stratification were informed by current CHB management guidelines [16-18] recommending antiviral treatment according to HBV DNA levels and HBeAg status. For HBeAg positive patients, treatment is recommended for viral loads of ≥ 10^5 ^copies/ml (approximately 20,000 IU/ml), while for those who seroconverted (became HBeAg negative), treatment is recommended for viral loads, of ≥ 10^4 ^copies/ml (approximately 2,000 IU/ml).

We used ALT levels of 1.5 times ULN to define high risk patients, in line with current recommendations [16-18]. In the absence of published data on ALT level distribution in people with CHB, we have estimated the proportion of patients with high viral load and high ALT using clinical data from the main teaching hospital in the pilot area. In the base case, we estimated that 50% of those patients with high viral loads also have elevated ALT and carried out sensitivity analyses around this estimate, assuming the proportion to be as low as 20%, or as high as 90%.

To estimate the size of the eligible population, we used data provided by the Australian Bureau of Statistics 2006 National Census on the number of local residents born in China, Hong Kong and Vietnam aged ≥ 35 years. We applied HBV sero-prevalence data on these numbers, based upon epidemiological estimates from the respective countries of birth [19].

We estimated the proportion of people in different CHB-related disease stages over time, using the assumptions of our Markov model published elsewhere [20]. Modelled disease stages included: CHB without cirrhosis, CHB with cirrhosis, CHB with liver failure, CHB and HCC, spontaneous HBsAg clearance, CHB-related death (due to liver failure or HCC) and death from other causes. The model structure and variables were informed by the available literature on the epidemiology and natural history of hepatitis-B related HCC [21-23] and by previously published models of CHB disease progression and treatment [24,25] Table 1 summarises the key assumptions of the model and Additional file 1 provides supplementary details about the model.

**Table 1:** General assumptions of the Markov model of HCC prevention

Assumption	How they were addressed and rationale
Recruitment of *B Positive *participants	Target population age ≥ 35, HBsAg + ve for ≥ 6 months, born in China, Hong Kong, Vietnam

Contact testing and immunisation	Not factored into the model

Seroprevalence data in target populations	Data provided by Nguyen et al [19]
	• 10.7% for people born in China
	• 10.5% for people born in Vietnam
	• 7.7% for people born in Hong Kong

Initial testing to confirm CHB	Not factored in GP consultation calculations

Program participation rates	Base case assumption is 25% of eligible people enrolled

Follow up requirements	• Routine surveillance arm: 2 GP appointments/year
	• Enhanced HCC surveillance arm: 2 GP appointments/year
	• Interferon treatment: 6 specialist appointments/year
	• Entecavir treatment (includes those with liver failure): 4 specialist appointments/year
	• Patients with HCC: assumed monthly follow up

Viral load distribution by age	Based upon REVEAL study, [22] as participant profile largely matches that of *B Positive *participants

ALT cut-off levels	ALT≥ 1.5 × ULN triggers further evaluation in the absence of clinical data; ULN differentiated by participant age

Progression rates through disease stages	Constant over time

Patients with high VL and abnormal liver function	30% receive first line interferon for 12 months
	• 30% seroconvert and receive no further treatment
	• 70% commence entecavir the following year
	70% receive entecavir as first-line treatment
	• 20% seroconvert in the first year of entecavir treatment and receive no further treatment
	• 80% continue lifelong entecavir

Patients with liver failure	All receive entecavir

VL: viral load

ALT: alanine aminotransferase

HBsAg: hepatitis B surface antigen

We factored in progressive recruitment over the first three years of the program and estimated a program participation rate of 25%, informed by the experience of the well-established New Zealand HBV screening programme, which screened 27% of the eligible (high risk) population [26]. We carried out sensitivity analyses, using a 10% participation rate as the lower bound, based upon a 8.8% HBV screening rate among Vietnamese migrants living in Eastern USA [27] and 65% as an upper bound, based upon the participation rate of Vietnamese women in cervical cancer screening in Central Sydney [28].

We used the model to determine the number of program participants in various CHB disease states and estimated the number of additional annual GP consultations required, i.e. we did not factor in consultations required for establishing a CHB diagnosis. We calculated the number of specialist visits conservatively, assuming that patients referred for specialist management receive treatment with interferon or entecavir. We assumed that 30% of people eligible for treatment would receive weekly interferon treatment for one year and that this would require 6 specialist consultations per year per patient. We estimated 4 specialist visits per year for the 70% of people on treatment receiving entecavir. We assumed that patients who become HBeAg negative (seroconvert) return to GP-based follow-up and that all those who are HBeAg positive, or have decompensated liver disease receive lifetime treatment with entecavir. We assumed a high adherence to treatment, based upon data derived from the area's largest teaching hospital (88% compliant with surveillance, 97% compliant with treatment (J George, personal communication). We conducted a sensitivity analysis, estimating that 60% of specialist referrals actually saw a specialist doctor, based upon available data from the Netherlands [29].

We calculated the number of liver US examinations likely to be required annually, by assuming that all people followed up in the enhanced CHB surveillance and specialist-led surveillance arms require biannual examinations.

We calculated the number of GP, specialist, and US appointments needed to manage CHB in this cohort and compared them to those required in the absence of the *B Positive *program (the 'baseline' scenario).

The number of GPs practising in the program area was provided by the local Division of General Practice. Approximately 330 General Practitioners are registered with the Liverpool Division (Sally Maspero, personal communication) and as a small number of GPs may not be registered with the local Division, we estimated a total primary care workforce of 350 GPs. An estimate of the number of liver specialists and gastroenterologists practising in the area was provided by the Gastroenterological Society of Australia. Their estimate of 28 local specialists was rounded off to 30 in the analysis. Due to diverse patterns of practice among radiologists with regards to diagnostic liver US (some practices offer this service routinely, others not at all), we calculated the number of US examinations required annually, but not the number of radiologists this could involve.

While we modelled costs and health outcomes over a 50-year period [20], in this paper we are focusing on health services demand in the first year after recruitment is completed, which is year 4 of the program.

## Results

Our findings suggest that in south-west Sydney approximately 8,300 people have CHB infection, with approximately 6,000 of them having been born in high prevalence countries; the latter represent the target population for the *B Positive *program (see Table 2).

**Table 2:** Estimated numbers of chronic hepatitis B cases in *B Positive *project target areas*

Number of people in the target population/estimated HBV seroprevalence	People born in Vietnam/10.5%	People born in China/10.7%	People born in Hong Kong/ 7%	Total in target countries of birth	All others/ 0.8%	Total CHB cases, irrespective of country of birth
**Total CHB cases, all ages**	5,044	2,625	237	7,905	3,814	11,720

**CHB cases, aged ≥ 35**	**3,544**	**2,100**	**161**	**5,805**	**2,550**	**8,355**

*Target areas: Fairfield, Liverpool, Bankstown, Canterbury and Auburn are Local Government Areas in the Greater Sydney metropolitan area

Target population: people born in Vietnam, China and Hong Kong in the program target area

We estimate that in the first year after cohort accrual, over 1,200 people would require medical follow up, with approximately 750 needing routine CHB surveillance, 250 enhanced CHB surveillance and just over 200 specialist-led surveillance and treatment. (See Table 3)

**Table 3:** Estimated annual GP, specialist and liver ultrasound appointments required in year 4 of the program and sensitivity analysis of incremental service utilisation, compared to baseline (no program)

Treatment group	Number of people*	Annual appointments related to CHB surveillance and/or treatment		
		
		GP appointments (averaged per GP)	Specialist (averaged per specialist)	Ultrasound
**Routine Surveillance **(GP-led)	745	1,303		

**Enhanced Surveillance **(GP-led)	263	460		263

**Interferon **(Specialist-led)	8		44	7

**Entecavir **(Specialist-led)	201		781	195

Other**	236		18	8

**Total cohort**	1,452	1,763	843	473

**Baseline **(no program)	1,452	28	84	54

**Incremental service utilisation (over no program)**				

**At 25% program uptake**	0	**1,734** *(5.0)*	**759** *(25.3)*	**419**

**At 10% program uptake**	0	**694** *(2.0)*	**304** *(10.1)*	**167**

**At 65% program uptake**	2	**4,509** *(12.9)*	**1,974** *(65.8)*	**1,089**

**With viral load cut-off of 2000 IU/ml at all ages in program**	0	**1,648** *(4.7)*	**961** *(32.0)*	**557**

**If ALT cut-off varies, so 20% have high ALT**	0	**1,908** *(5.5)*	**274** *(9.1)*	**380**

**If ALT cut-off varies, so 90% have high ALT**	0	**1,475** *(4.2)*	**1,370** *(45.7)*	**427**

**If age at enrolment is ≥ 40 years (instead of 35)**	0	**1,327** *(3.8)*	**616** *(20.5)*	**344**

*Estimated at Year 4, after recruitment has been completed

** other = HCC diagnoses (n = 2); HbeAG seroconversions (n = 47); program dropouts (n = 187)

In year 4 of the program, we estimate that two participants will receive a HCC diagnosis and that approximately 50 people will undergo HBeAg seroconversion, either spontaneously, or after drug treatment. Approximately 190 participants will discontinue follow-up by the end of year 4.

Table 4 presents data on the incremental use of services over the life of the program, at 5-year intervals.

**Table 4:** Estimated incremental use of GP, specialist, and ultrasound services appointments over the life of the program

Year	*Number of people in cohort*	Incremental appointments per program year		
		
		GP	Specialist	Ultrasound
**Year 1**	*290*	406	169	99

**Year 2**	*875*	1,165	483	282

**Year 3**	*1462*	1,808	758	436

**Year 4**	*1,452*	1,734	759	419

**Year 5**	*1,442*	1,628	765	402

Year 8*	*1,404*	1,346	718	379

**Year 13**	*1,342*	1,030	561	291

**Year 18**	*1,257*	776	433	213

**Year 23**	*1,150*	574	340	158

**Year 28**	*1,020*	413	266	116

**Year 33**	*861*	284	200	82

**Year 38**	*683*	183	142	56

**Year 43**	*499*	109	93	36

**Year 48**	*331*	58	57	21

* Assumes recruitment is completed in year 4

For years 6-50, we calculated the mid-point figures for each 5 year period

Approximately 1,700 additional GP appointments would be needed in the first year after program recruitment is complete. This translates into about 5 additional appointments a year for each of the 350 GPs in the area. We estimated that an additional 760 specialist appointments would also be needed, or about 25 appointments per specialist. The cohort would also require approximately 420 US examinations.

**Figure 2:**
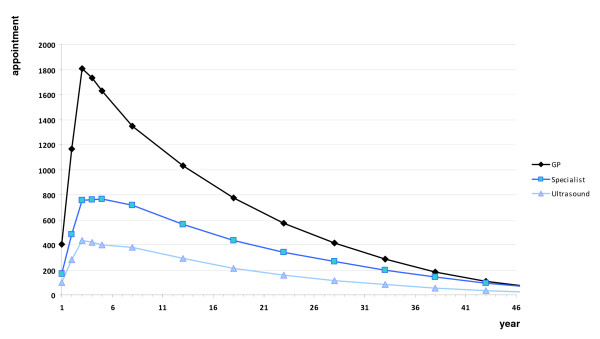
**Estimated incremental GP, specialist, and ultrasound services per year over the life of the program**.

Figure 2 illustrates the number of additional consultations with general practitioners, specialists and the number of ultrasound examinations expected to be required over the duration of the project.

### Sensitivity Analyses

While the base case assumed a 25% program uptake, we also estimated the impact lower or higher recruitment would have on service utilisation. (See Table 3) Assuming a 10% program uptake, some additional 700 GP appointments and 300 specialist appointments would be required. This translates into 2 additional appointments for each GP and 10 extra appointments for each specialist per year. Assuming a 65% program uptake, approximately 4,500 more GP appointments and 2,000 more specialist appointments would be required in year 4. This translates into approximately 13 additional appointments per GP and 66 appointments per specialist per year.

Using a lower ALT cut-off level had the greatest impact on health service utilisation. Assuming that 90% of enrolees have ALT levels that require antiviral treatment (as compared to 50% in the base case), annual consultations per specialist would increase from 25.3 (at baseline) to 47.7. Assuming that 20% of participants have ALT levels requiring antiviral treatment would lead to a slight increase in GP services (from 5.0 at baseline to 5.5) and a significant reduction in the need for specialist services (from 25.3 to 9.1 per specialist) - see Table 3.

Using a lower cut-off for viral load level (2,000 IU/ml) for all enrolled patients, regardless of their age, leads to a slight reduction in the number of GP consultations (from 5.0 to 4.7 per GP), but a significant increase in specialist utilisation (from 25.3 to 32.0 per specialist).

Increasing the age at program enrolment from 35 to 40 years (age when guidelines suggests that HCC screening should commence) [23] reduces both the demand for GP services (from 5.0 at baseline to 3.8 visits per GP) and for specialist consultations (from 25.3 to 20.5 per specialist).

Sensitivity analyses on the risk reduction conferred by antiviral treatment on progression from CHB to cirrhosis, cirrhosis to liver failure and liver failure to death found that varying assumptions had practically no effect on the workload of GPs, specialists, and on the number of ultrasound examinations required (see Additional Files 1, 2, 3, 4 and 5).

## Discussion

Our findings suggest that a program of liver cancer prevention in a population at risk of approximately 6000 people will lead to approximately 1700 additional primary care appointments, 750 specialist appointments and 400 US examinations, assuming a 25% participation rate. Almost 80% of this cohort would remain under the care of their primary care providers, and given the total number of GPs in the area, the average GP would need to provide just 5 additional appointments per year for *B Positive *patients. However, as the distribution of CHB patients among practices is uneven, some GPs may face a significant increase in workload if they care for large numbers of CHB patients. Assuming an average of 1.73 incremental GP appointments per person enrolled in the program, a GP caring for 100 CHB patients would need to accommodate 173 additional patient consultations each year. Therefore we estimate that overall the *B Positive *program is likely to have a significant impact upon the demand for primary care services in the program area.

Our analysis was purposefully restricted to an area of high CHB prevalence, in order to estimate the greatest potential impact on health service utilisation in the "worst case scenario" in terms of demand for services. In areas with lower HBV seroprevalence, such a program would have a lesser impact on the number of primary care consultations required.

Our results suggest a significant increase in demand for specialist services, amounting to approximately 750 additional specialist consultations, or approximately 25 additional consultations per provider per year. As approximately 200 patients enrolled in the *B Positive *program would need specialist care, this would translate into approximately 7 new CHB patients referrals per specialist. As referrals to specialists are likely to be unevenly distributed, some specialists could face a significant increase in demand for their services, compared to their current practice.

The 400 additional radiology appointments required are likely to be readily accommodated, as they would be distributed among a large number of private radiology providers in the area.

We assumed a modest uptake of the program (25%) among people at risk, but a high level of adherence to treatment once enrolled, based upon the experience of a teaching hospital in the program area. Lower treatment adherence would reduce the number of patients seen by medical practitioners while also limiting the health benefits associated with the program.

The *B Positive *program development was predicated upon the assumption that a substantial proportion of CHB patients can be managed at the primary care level, provided GPs are aware of current best-practice management guidelines. The *B Positive *risk stratification algorithm provides GPs with an alternative to specialist referral for patients at lower risk of HCC, who do not require antiviral therapy or liver cancer screening. Our economic model proved that the program is cost-effective [20] and the current study suggests that its impact upon the GP workforce is manageable, within currently available resources.

### Study limitations

In the absence of Australian HBV seroprevalence studies, we used CHB prevalence estimates based upon studies in Asian migrant populations in the UK, Canada and US [30]. In the absence of local data on participation rates in a hepatitis B screening and follow up program, we assumed similar participation rates for different migrant groups, although data from New Zealand suggest that significant differences exist with regards to CHB screening preferences by age and ethnicity [26].

As no consensus exists on ALT levels for treatment initiation, and as elevated ALT levels in patients with CHB may also be due to overweight or obesity (present in over 60% of Australian adults) [31], we estimated that approximately 50% of people with elevated viral loads also have elevated ALT levels. We used data derived from the REVEAL study to estimate the rate of malignant transformation by level of viral load [22], but acknowledge that factors such as viral genotypes, smoking, obesity, alcohol consumption and host genetic polymorphisms [32-36] may also play an important role. The total number of specialist consultations is contingent upon the number of referrals, as well as the actual viral load and ALT levels present in the target population. In the absence of local data, these were inferred from other studies, or specialist opinion, so the numbers of episodes of care delivered may be at variance from the ones estimated by this paper.

Data collected through the *B Positive *project suggests that some local GPs have more than 100 patients with CHB in their practice records. We acknowledge that in areas with a large Asian-born population, a shortage in the number of local GPs and especially specialists would present significant challenges to program implementation. As approximately two thirds of GP practices are using practice management software programs that can support an electronic clinical decision making system, we are currently developing a GP desktop software module to facilitate patient recall and management; this would assist GPs to better manage the additional workload imposed by the *B Positive *program upon participating practices.

We assumed that most of the episodes of care take place in the public health sector, but have insufficient information on the current contribution of the private health sector to CHB management. As liver cancer is concentrated among populations of relative disadvantage, we assumed that the contribution of the private sector would be limited, but the validity of this assumption has not been tested. Additionally, equity implications of excluding the private sector from public health programs merit careful consideration. The mobilisation of private sector specialist resources could provide an expansion of specialist capacity in the short term, largely through the engagement of a wider pool of specialist gastroenterologists. Active involvement of government in contracting for services and/or staffing and in enabling community clinics to support their employment may facilitate private sector involvement, without shifting the financial burden to relatively disadvantaged patients and families.

In its 2005 report, the Australian Productivity Commission highlighted the need for an appropriate workforce in terms of structure and skills mix, to address current and emerging health care needs of Australians [37]. Under existing workforce arrangements, an increased capacity to manage CHB is conditional upon increasing the specialist workforce. This can be achieved through training additional specialists, overseas recruiting of doctors, or by using technology allowing the automation of some of the routine surveillance and follow up tasks. Creating shared decision support and review systems could provide a seamless transition from primary to specialist care, as dictated by changes in patients' disease states. In the future, we envisage that some of the components of the patient care pathway currently serviced by specialists (such as follow up of patients receiving antiviral medications) could be carried out in primary care, as is already the case for other blood borne infections, such as HIV and hepatitis C. Using practice nurses for CHB screening, outreach and follow up could provide additional resources to facilitate the follow up of low risk, routine CHB cases in primary care, but in the absence of data on their actual or projected numbers and the practice locations, it can only be hypothesised that this emerging segment of the workforce may have a significant future role in CHB surveillance and liver disease prevention.

## Conclusions

While a targeted screening and treatment program of CHB among people born in high HBV prevalence countries can be cost-effective in an Australian setting, program implementation is contingent upon the health system's ability to offer follow up and treatment to all those needing it. While the burden of additional consultations appears manageable at primary care level, a significantly higher demand for specialist care poses significant challenges to the health system. To address this challenge, health planners will need to explore a range of options, to ensure that medical specialists are available to take on this additional work. A re-evaluation or re-design of some of the health workforce roles may be required, to effectively address the challenge imposed by the growing numbers of CHB and HCC diagnoses expected in Australia over the next 20 years.

## Abbreviations

**HCC**: hepatocellular cancer, **HBV**: hepatitis B virus, **CHB**: chronic hepatitis B, **LGA**: local government area, **RACGP**: Royal Australasian College of General Practitioners, **HBsAg**: hepatitis B surface antigen, **ALT**: alanine amino transferase, **HBeAg**: hepatitis B e antigen, **VL**: viral load, **GP**: general practitioner;

## Competing interests

The authors declare that they have no competing interests.

## Authors' contributions

MCR conceived and coordinated the study, participated in its design, contributed to data analysis and drafted the manuscript; MQK participated in study design, developed the economic model, performed the statistical analysis and helped draft the manuscript; JG participated in study design and helped draft the manuscript; KH participated in study design and statistical analysis and helped draft the manuscript; ST participated in study design and data collection; ML participated in study design and data collection; NP participated in study design; AGP participated in study design and helped draft the manuscript; ALL AUTHORS approved the final version of the paper

## Pre-publication history

The pre-publication history for this paper can be accessed here:

http://www.biomedcentral.com/1472-6963/10/215/prepub

## Supplementary Material

Additional file 1 **Details of the economic model**. A detailed description of the economic model, its assumptions, main findings and data sources. Click here for file

Additional file 2 **Table S1: Epidemiological transition probabilities for people with chronic hepatitis B managed under current practice and under the modelled HCC prevention program**. CHB progressions states modelled using 6 discrete states; transition probabilities presented, together with data sources. Click here for file

Additional file 3 **Table S2: Cost of resources used up for routine hepatitis care, CHB surveillance and HCC prevention**. Costs calculated for all tests and services utilised (except palliative care)-unit costs calculated; expert opinion used where costing not available (cost of chemoembolisation and viral load assay). Click here for file

Additional file 4 **Table S3: Utility weights used in the economic model**. Lists age-specific weightings used in the model and their derivation. Click here for file

Additional file 5 Table S4: Sensitivity analysis on the estimated effects of the program on disease progression and number of medical appointments in year 4 of the program*. Sensitivity analysis of assuming different rates of reduction of cases progressing from CHB to cirrhosis, cirrhosis to liver failure and liver failure to death. Click here for file
