# Supplementary material for: Using a population-based approach to prevent hepatocellular cancer in New South Wales, Australia: effects on health services utilisation
Source: BMC Health Serv Res. 2010 Jul 21;10:215. doi: 10.1186/1472-6963-10-215 (PMC2918596; doi:10.1186/1472-6963-10-215)
Supplement: Additional file 1 — Details of the economic model. A detailed description of the economic model, its assumptions, main findings and data sources. [file 1472-6963-10-215-S1.DOCX]

# Additional file 1:

# details of the economic model

We modelled the disease progression of chronic hepatitis B (CHB) using six discrete disease states and assuming an enrolled population of approximately 1,500 people, based on the estimated cases of CHB in the program areas in Sydney, Australia and the estimated take-up rate in the target population. We chose these areas because of their concentration of populations born in China, Hong Kong, and Vietnam – three regions where HBV infection is endemic and which contribute to a significant number of immigrants to Australia. In this population, we modelled progression through different CHB disease states and estimated the impact of a program of CHB screening, surveillance and treatment for those at high risk of developing HCC, based upon age, viral load and ALT level.

We modelled total healthcare costs specific to CHB and its sequelae, regardless of payer or beneficiary; the only specific exclusion was the cost of palliative care. We tracked total cases of cirrhosis, liver failure and HCC, as well as deaths averted and calculated quality-adjusted life years (QALYs), in order to assess the health outcomes and benefits of the program. We estimated both costs and benefits over 50 years for all those who joined the program (even if they drop out of the program later). We assessed outcomes annually in years 1-5 of the program and five-yearly thereafter; we assumed that interventions take place annually at the start of year or upon joining of the program if recruited in that period. We discounted all future costs and benefits to the present at 5%, (consistent with government recommendations), and to allow comparisons to other papers describing research on the same topic.

We developed the model in Microsoft Excel, as this is a readily accessible platform for modelling, also used by other groups working in HBV modelling, for example Hutton et al [38].

Data and sources

We estimated model variables including progression rates and treatment effectiveness based upon estimates from the medical literature, or where direct published estimates were unavailable, expert estimates. As we modelled entecavir as the main antiviral treatment, in the absence of long-term studies on the drug’s effectiveness, we used proxies and the limited available data to estimate this treatment’s effectiveness.

For costs, we used Australia’s Medicare schedule fees to estimate the costs for screening, surveillance, and treatment elements. We used sensitivity analysis to model ranges of estimates for key variables, including treatment effectiveness and costs of particular treatments.

Disease states, ageing, and progression

We modelled CHB disease progression using six discrete states: CHB without cirrhosis; CHB with cirrhosis; CHB with liver failure; CHB with HCC; CHB clearance and death. In order to track health outcomes, we separately tracked in the model deaths due to CHB-related causes (i.e., liver failure or HCC) from those of all other causes. Due to the small number of transplants available, we did not model transplant as a possibility- see **Additional file 2,** **Table S1**

In the program scenario, we modelled one cohort in the program (‘in treatment’) and one cohort out of the program (i.e., drop-outs from the program); we did not individually model sub-cohorts by viral load, due to a lack of available data on progression rates for such sub-cohorts. Thus the estimated reduction in disease progression rates was estimated for and applied to the entire cohort ‘in treatment’ – assumed to be a blended average of those with high viral load receiving drug treatment and those with low viral load not receiving treatment (who would be expected to have a slow progression rate).

We used viral load and ALT levels to determine the patient subgroup where drug treatment would be indicated. We estimated that 30% of people eligible for treatment had interferon weekly for 12 months as first-line treatment and assumed that 30% of this group would achieve sustained viral suppression and have no subsequent treatment. The remainder received entecavir therapy (0.5 mg daily) from year 2 onwards. We estimated that 70% of people eligible for treatment received entecavir, with 20% assumed to seroconvert during the first year of treatment (and then receiving no further treatment); the remainder continued entecavir therapy indefinitely. We also modelled the transition from low to high viral load, in order to estimate the number of people in the cohort who would be likely to start drug treatment in each year. We modelled this, based on expert opinion, at 5% per year who would transition from low to high viral load. We did not model different rates of disease progression for those with varying levels of viral load or ALT, as we were not aware of data that would allow us to model these outcomes.

We modelled progression for 10-year age cohorts for all ages that enter the program (ages 35 and up). We assumed that those who entered the program were equally distributed amongst the 10 years within each age band. At each change in period, we aged the group by the appropriate amount, moving some to a different age band, as necessary. While we specifically modelled these age cohorts by gender, due to a lack of available data, we modelled the same progression rates for both males and females for all ages in the program, with the exception of the rate of death from other causes, for which we used age- and gender-specific rates. We assumed that all participants died at age 90, if not before.

Treatments and treatment effectiveness

For those with CHB and cirrhosis, we assumed that all those with high viral load and high ALT would receive drug treatment. We chose the treatments to model based upon expert opinion, as well as on what is current clinical practice. The treatments we modelled were: Interferon (Peginterferon Alfa-2a 180 micrograms once-weekly) or entecavir (0.5mg once-daily). We assumed that all those with liver failure receive entecavir, regardless if they were enrolled in the program or not.

For those diagnosed with HCC, we assumed that a percentage would be eligible for resection; those not eligible for resection would receive one year of treatment with chemoembolisation and radiofrequency ablation. As noted above, we did not include either transplant or palliative care in cost calculations.

Screening & surveillance protocol

We modelled the initial screening protocol based on basic tests used to identify CHB and tests for viral load and liver function, in order to assess the patient’s risk level. Those testing negative would be discharged from the program with only the initial HBsAg tests for these patients included in program costs. In order to assess the level of risk for each enrolled patient, all participants have a determination of their viral load (HBV DNA) and liver function (ALT). Those with high viral load and high ALT (i.e. at high risk) also have AFP and ultrasound screening for HCC, as well as a liver biopsy. We included the incremental costs of the GP appointments for testing and discussion of test results, and for those deemed high risk, also the cost of a specialist appointment.

After initial screening, patients are directed into one of two types of surveillance protocol: ‘routine care’ and ‘enhanced surveillance.’ All those with high HBV viral load, regardless of ALT level, are streamlined into enhanced surveillance. Both routine care and enhanced surveillance involve six-monthly follow up and are GP-led. Routine care includes three hepatitis B blood tests (HBsAg, HBeAg, and HBV DNA) as well as a liver function test (ALT) with two GP appointments (one for testing and one for discussion of results). Enhanced surveillance also includes liver ultrasound and AFP, to screen for HCC.

In addition, those at high risk are candidates for drug treatment; as noted above, we assumed for modelling purposes that all of these patients received drug treatment. For those under drug treatment, we also included the costs for regular specialist oversight at a frequency agreed upon by the clinicians on our panel.

In order to define ‘high risk’ we relied on a combination of the results published in the REVEAL study and expert clinical judgment. It was our expert clinicians’ judgment that we should vary the cut-off level based upon age; based on their opinion and the REVEAL data, we chose 20,000 IU as the cut-off for those aged 35-49 and 2,000 IUfor those aged 50 and above. We included ALT as a further guideline for defining ‘high risk’ and chose a level of 1.5xULN as the cut-off, estimating that 50% of those with high HBV DNA levels would also have high ALT. We estimated this proportion based on a combination of clinical data from a hospital in Sydney, REVEAL data, and expert judgement as there was little directly applicable published data.

Costs

We included the costs for all tests and healthcare for the cohort entering the program related to CHB and its sequelae, except for palliative care costs. We also included these costs for those who drop out of the program at some point, as well as the testing costs for those who are screened, but are negative for HBsAg. In order to focus our assessment of cost-benefit on the program, we excluded both costs and benefits for any cases of HCC that might be detected at the initial screening and program entry. We did not include any testing or treatment costs for close contacts of program participants; rather, we assumed these to be included in those we recruit.

Costs for nearly all elements were obtained from Australia’s Medicare Benefits Schedule (MBS) or Prescription Benefits Schedule (PBS). Costs not available from either the MBS or PBS included: the HBV DNA test, chemoembolisation and liver resection. We estimated the HBV DNA test would cost AU$120 based on current cost estimates in Australia; the costs for chemoembolisation and liver resection were estimated based on hospital data from a Sydney hospital which will be the main hospital of treatment for the program – these costs were modelled at AU$314 and AU$27,196 (including both cases with and without complications) respectively. See **Additional file 3: Table S2)**

Epidemiological variables

We assumed that the prevalence of chronic hepatitis B in Sydney amongst a population born in a particular country would be similar to that person’s country of birth and used these figures to estimate each of these populations’ median prevalence. We used data from the Australian Bureau of Statistics’ 2006 census in order to estimate the size by gender and by age of each of these populations in our focus geographies. In order to estimate total prevalence we ‘curved’ the overall prevalence rate by age and gender, based on the age and gender curve found in New Zealand’s hepatitis B program. We then combined these estimates – along with estimates of overall prevalence in lower-risk Australian populations – in order to estimate the total number of CHB cases in the focus geographies. We used a combination of proxies in order to estimate the starting number of cirrhotics in the program.

Health outcomes

We measured discounted QALYs experienced by people in the cohort, whether they remained in the program or not, as well as: the number of cases of cirrhosis, liver failure and HCC; and the total number of CHB-related deaths. We assumed that each life-year lost as a result of CHB-related death represents one lost QALY; for other health states we used age-specific utility weightings based on a published study in the UK Health Technology Assessment on the cost-effectiveness of adefovir and pegylated interferon for treatment of chronic hepatitis B. In applying these weightings, we chose the more conservative figure where there was a choice between two applicable figures – for example, we used the weighting for chronic hepatitis B for all those in the CHB state, not applying the (higher) weighting for those seroconverted, although some in our cohort would do so. We assumed no CHB-related utility loss for those who clear CHB. (See **Additional File 4: Table S3**)

Program characteristics

We included costs of program administration, recruitment, and retention in our modelling; we estimated these figures were based on the approved budget for the program. We modelled recruitment and retention rates based on our own estimates. As recruitment focus is on the populations born in China and Vietnam , we assumed that we would reach a 25% take-up rate amongst this target population; for the purposes of this paper, we modelled zero recruitment outside our target population. For the purposes of this paper, we modelled recruitment to take place in the first three years of the program (20% of total in Year 1; 40% of total in Year 2; and final 40% of total in Year 3), with no steady-state entry thereafter.

We estimated adherence rates to model for treatment and surveillance based on unpublished data from a study in Westmead hospital, where many of the program participants will be treated; we modelled an 88% percent adherence to surveillance and 97% to drug treatment.

Baseline

In order to calculate the incremental costs and benefits of the program we modelled the health outcomes and costs for the cohort over 50 years of the program (including those who would drop out) and without it, and then calculated the difference. We used a variety of sources to estimate the baseline number of people who would receive drug treatment for CHB, and liver resections. For drug treatment, we estimated the percentage of CHB patients in New South Wales currently receiving drug treatment for CHB based on our estimate of the total number of cases of CHB in New South Wales (approximately 55,000) and government data on the total number of prescriptions in the year ending June 30, 2007 of a range of hepatitis B treatment drugs. This percentage was 0.86%; for modelling purposes, 1% was used to estimate the percentage of people in the cohort who would receive drug treatment without the program. We assumed that of those people treated outside the program, 50% would receive some surveillance without the program; we modelled this surveillance as including HBsAg, HBeAg, ALT, and AFP testing and ultrasound. We assumed that all liver failure cases receive drug treatment, whether in or out of the program.

For liver resections, we assumed that, because of the program’s regular surveillance, cases of HCC would be identified earlier and thus, a higher proportion would be resectable. We used the results from Yang’s 1997 study, conducted in China, which showed that with six-monthly AFP and ultrasound, the percentage of HCCs resectable was 89.5% as compared to 11.1%.[39]

***Recruitment costs***

In this setting, a well-defined geographic area and target population, we factored in a modest cost for marketing/ recruitment of participants: AUD 50,000 for media costs & advertising plus $200 per GP in the area for education and recruitment. We also included program administrative costs of $30,000 in fixed costs plus $30-70 per patient per year, depending on the year of the program. When modelling the entire Sydney metropolitan region and the state of NSW, we included a larger amount for marketing (AUD100, 000 and AUD1 million respectively).

The pilot is based in an area of geographic and ethnic clustering of CHB cases in the State, where a substantial proportion of primary care practitioners speak one of the relevant languages used by the target population. The pilot program is underpinned by a community engagement and general practitioner information program and is supported by community liaison officers speaking Vietnamese, Cantonese and Mandarin, who can interpreter provide assistance. We acknowledge that language and other barriers represent significant challenges for recruitment .

***Model cutoffs for the surveillance and treatment programs***

Our treatment cut-offs with regards to **viral load** were informed by algorithms developed by groups charged with guideline development. They are generally in agreement that threshold levels of HBV DNA should be individualized depending on whether patients are:

- HBeAg positive, when treatment is recommended for viral loads ≥ 10^5^ copies /ml, or approximately 20,000 IU /ml
- HBeAg negative, when recommended threshold levels of HBV DNA are one order of magnitude lower, with treatment suggested for VL≥ 10^4^ copies /ml, or approximately 2,000 IU /ml. [16; 17; 18]

The lower **age limit** in our study (patients aged ≥35 years) was informed by the first large study examining spontaneous HBeAg sero-conversion in over 3000 Chinese participants. The median age at HBeAg sero-conversion was 34.5 years and sero-conversion was much more likely to occur in those with elevated ALT levels. [40] In a study examining the prevalence of significant histology in a patient population with mildly elevated serum ALT levels, Tsang et al found that age >35 years (together with being male and having higher ALT levels) best predicted significant histology on multivariate analysis. [41]

Our model therefore took into account a patient’s viral load as well as age (ages 35-49 years and ≥50 years). The 35-49 age group was chosen because it is likely that a significant proportion in this age group would have active liver disease and would therefore benefit from screening for HBV infection.

At the older age limit (of ≥50), a sufficient proportion of patients would be at risk of developing cirrhosis (based upon a progression rate of 2.1% per year, as suggested by Liaw [17], placing them at an increased risk of hepatic decompensation and liver cancer. Based upon the recommendations of the American Gastroenterology Association Institute we used a lower viral load (≥ 10^4^ copies /ml or approximately 2,000 IU /ml) for treatment initiation in the older age groups. [16]

We acknowledge that the REVEAL study shows that an elevated HBV DNA is the best predictor of HCC, but all current guidelines (including the European Association for the Study of Liver Disease, The Asian Pacific Association for the Study of the Liver, the American Gastroenterological Association Institute, the AASLD Guidelines and the UK National Institute for Health and Clinical Excellence guidelines), recommend taking into consideration both **viral loads**, as well as **ALT elevation** when deciding on initiation of therapy. Essentially they recommend consideration of therapy if ALT is elevated, and continuing observation if ALT is normal and our model took into account the above recommendations.

We used 1.5 times ULN as a cut-off for what represents an abnormal ALT, which is lower than what is currently recommended by most guidelines. While we are aware of studies emphasizing treatment at even lower ALT levels (based upon findings that patients with chronic infection and persistently normal ALT levels can be at an increased risk of fibrosis), [41; 42] we did not incorporate this in our model. As risk stratification algorithm will be carried out by primary care providers (who largely prefer to follow current guidelines), we decided to adopt a level of ALT ≥1.5 times ULN as the trigger for referral and therapy, as this is more congruent with current recommendations. Without published data on the distribution of ALT level based on viral load, we estimated the proportion of patients with high viral load and high ALT based on expert opinion and data from the main hospital in the pilot area; in the base case of the model, we estimated 50% of those patients with high viral load will also have elevated ALT.

At baseline we assumed that the risk reduction with treatment is 90% in the progression from CHB to cirrhosis and cirrhosis to liver failure. The risk reduction in reducing progression from liver failure to death is not influenced by treatment, as all people requiring treatment are assumed to receive it under current conditions, irrespective of whether they are enrolled in the program or not. Assuming either a 50% or a 70% reduction in the risk of progression from CHB to cirrhosis and from cirrhosis to liver failure did not materially impact on the number of doctor appointments, or US examinations. We also varied the assumptions around the risk reduction from liver failure to death, which was 0% in the base case. Assuming either a 10% risk reduction, or a 50% risk reduction did not have a differential impact on the need for medical services in this cohort- see **Additional file 5: Table S4.**

# REFERENCES:

1. Alam N, Chen W, Baker D, and Bishop J. 2009. **Liver Cancer in New South Wales**. C. I. NSW. Sydney: Cancer Institute NSW.

2. Amin J, Dore GJ, O'Connell DL, Bartlett M, Tracey E, Kaldor JM, and Law MG. 2006. **Cancer incidence in people with hepatitis B or C infection: a large community-based linkage study**. *J Hepatol* **45**(2): 197-203.

3. Beasley RP, Hwang LY, Lin CC, Stevens CE, Wang KY, Sun TS, Hsieh FJ, and Szmuness W. 1981. **Hepatitis B immune globulin (HBIG) efficacy in the interruption of perinatal transmission of hepatitis B virus carrier state. Initial report of a randomised double-blind placebo-controlled trial**. *Lancet* **2**(8243): 388-93.

4. Bosch FX, Ribes J, Diaz M, and Cleries R. 2004. **Primary liver cancer: worldwide incidence and trends**. *Gastroenterology* **127**(5 Suppl 1): S5-S16.

5. Gellert L, Jalaludin B, and Levy M. 2007. **Hepatocellular carcinoma in Sydney South West: late symptomatic presentation and poor outcome for most.** *Internal Medicine Journal* **37**(8): 516-22.

6. Dore G, Wallace J, Locarnini S, Desmond P, Gane E, and Crawford DH. 2006. *Hepatitis B in Australia: responding to a diverse epidemic*. Sydney.

7. Amin J, O'Connell D, Bartlett M, Tracey E, Kaldor J, Law M, and Dore G. 2007. **Liver cancer and hepatitis B and C in New South Wales, 1990-2002: a linkage study**. *Aust N Z J Public Health* **31**(5): 475-82.

8. Supramaniam R, O'Connell DL, Tracey E, and Sitas F. 2006. **Cancer incidence in New South Wales migrants 1991 to 2001**. Sydney: The Cancer Council NSW.

9. Zhang BH, Yang BH, and Tang ZY. 2004. **Randomized controlled trial of screening for hepatocellular carcinoma**. *J Cancer Res Clin Oncol* **130**(7): 417-22.

10. Wun YT and Dickinson JA. 2003. **Alpha-fetoprotein and/or liver ultrasonography for liver cancer screening in patients with chronic hepatitis B**. *Cochrane Database Syst Rev*((2)): CD002799.

11. Fung SK and Lok AS. 2004. **Treatment of chronic hepatitis B: who to treat, what to use, and for how long?** *Clin Gastroenterol Hepatol* **2**(10): 839-48.

12. Hache C and Villeneuve JP. 2006. **Lamivudine treatment in patients with chronic hepatitis B and cirrhosis**. *Expert Opin Pharmacother* **7**(13): 1835-43.

13. Lok AS and McMahon BJ. 2004. **Chronic hepatitis B: update of recommendations**. *Hepatology* **39**(3): 857-61.

14. National Centre in HIV Epidemiology and Clinical Research. 2007. **HIV/AIDS, viral hepatitis and sexually transmitted infections in Australia. Annual Surveillance Report**. N. C. i. H. E. a. C. Research. Sydney: National Centre in HIV Epidemiology and Clinical Research.

15. Tipper S and Penman AG. 2009. **The NSW HBV and liver cancer pilot program: an update on the ‘B Positive’ Project**. *Cancer Forum* **33**(2).

16. Keeffe EB, Dieterich DT, Han SH, Jacobson IM, Martin P, Schiff ER, Tobias H, and Wright TL. 2006. **A treatment algorithm for the management of chronic hepatitis B virus infection in the United States: an update**. *Clin Gastroenterol Hepatol* **4**(8): 936-62.

17. Liaw YF, Leung N, Guan R, Lau GK, Merican I, McCaughan G, Gane E, Kao JH, and Omata M. 2005. **Asian-Pacific consensus statement on the management of chronic hepatitis B: a 2005 update**. *Liver Int* **25**(3): 472-89.

18. Thomas HC. 2007. **Best practice in the treatment of chronic hepatitis B: a summary of the European Viral Hepatitis Educational Initiative (EVHEI)**. *J Hepatol* **47**(4): 588-97.

19. Nguyen VT, Razali K, Amin J, Law MG, and Dore GJ. 2008. **Estimates and projections of hepatitis B-related hepatocellular carcinoma in Australia among people born in Asia-Pacific countries**. *J Gastroenterol Hepatol* **23**(6): 922-9.

20. Robotin MC, Kansil M, Howard K, George J, Tipper S, Dore GJ, Levy M, and Penman AG. 2009. **Antiviral therapy for hepatitis B-related liver cancer prevention is more cost-effective than cancer screening**. *J Hepatol* **50**(5): 990-8.

21. Bolondi L, Sofia S, Siringo S, Gaiani S, Casali A, Zironi G, Piscaglia F, Gramantieri L, Zanetti M, and Sherman M. 2001. **Surveillance programme of cirrhotic patients for early diagnosis and treatment of hepatocellular carcinoma: a cost effectiveness analysis**. *Gut* **48**(2): 251-9.

22. Chen CJ, Yang HI, Su J, Jen CL, You SL, Lu SN, Huang GT, and Iloeje UH. 2006. **Risk of hepatocellular carcinoma across a biological gradient of serum hepatitis B virus DNA level**. *Jama* **295**(1): 65-73.

23. Sherman M. 2004. **Pathogenesis and screening for hepatocellular carcinoma**. *Clin Liver Dis* **8**(2): 419-43, viii.

24. Kanwal F, Gralnek IM, Martin P, Dulai GS, Farid M, and Spiegel BM. 2005. **Treatment alternatives for chronic hepatitis B virus infection: a cost-effectiveness analysis**. *Ann Intern Med* **142**(10): 821-31.

25. Shepherd J, Jones J, Takeda A, Davidson P, and Price A. 2006. **Adefovir dipivoxil and pegylated interferon alfa-2a for the treatment of chronic hepatitis B: a systematic review and economic evaluation**. *Health Technol Assess* **10**(28): iii-iv, xi-xiv, 1-183.

26. Robinson T, Bullen C, Humphries W, Hornell J, and Moyes C. 2005. **The New Zealand Hepatitis B Screening Programme: screening coverage and prevalence of chronic hepatitis B infection**. *N Z Med J* **118**(1211): U1345.

27. Ma GX, Fang CY, Shive SE, Toubbeh J, Tan Y, and Siu P. 2007. **Risk perceptions and barriers to Hepatitis B screening and vaccination among Vietnamese immigrants**. *J Immigr Minor Health* **9**(3): 213-20.

28. Lesjak M, Hua M, and Ward J. 1999. **Cervical screening among immigrant Vietnamese women seen in general practice: current rates, predictors and potential recruitment strategies**. *Aust N Z J Public Health* **23**(2): 168-73.

29. Mostert MC, Richardus JH, and de Man RA. 2004. **Referral of chronic hepatitis B patients from primary to specialist care: making a simple guideline work**. *J Hepatol* **41**(6): 1026-30.

30. Nguyen VT, Law MG, and Dore GJ. 2009. **Hepatitis B-related hepatocellular carcinoma: epidemiological characteristics and disease burden**. *J Viral Hepat* **16**(7): 453-63.

31. Cameron AJ, Welborn TA, Zimmet PZ, Dunstan DW, Owen N, Salmon J, Dalton M, Jolley D, and Shaw JE. 2003. **Overweight and obesity in Australia: the 1999-2000 Australian Diabetes, Obesity and Lifestyle Study (AusDiab)**. *Med J Aust* **178**(9): 427-32.

32. Calle EE, Rodriguez C, Walker-Thurmond K, and Thun MJ. 2003. **Overweight, obesity, and mortality from cancer in a prospectively studied cohort of U.S. adults**. *N Engl J Med* **348**(17): 1625-38.

33. Chen ZM, Liu BQ, Boreham J, Wu YP, Chen JS, and Peto R. 2003. **Smoking and liver cancer in China: case-control comparison of 36,000 liver cancer deaths vs. 17,000 cirrhosis deaths**. *Int J Cancer* **107**(1): 106-12.

34. Donato F, Tagger A, Gelatti U, Parrinello G, Boffetta P, Albertini A, Decarli A, Trevisi P, Ribero ML, Martelli C, Porru S, and Nardi G. 2002. **Alcohol and hepatocellular carcinoma: the effect of lifetime intake and hepatitis virus infections in men and women**. *Am J Epidemiol* **155**(4): 323-31.

35. Ni YH, Chang MH, Wang KJ, Hsu HY, Chen HL, Kao JH, Yeh SH, Jeng YM, Tsai KS, and Chen DS. 2004. **Clinical relevance of hepatitis B virus genotype in children with chronic infection and hepatocellular carcinoma**. *Gastroenterology* **127**(6): 1733-8.

36. White DL, Li D, Nurgalieva Z, and El-Serag HB. 2008. **Genetic variants of glutathione S-transferase as possible risk factors for hepatocellular carcinoma: a HuGE systematic review and meta-analysis**. *Am J Epidemiol* **167**(4): 377-89.

37. Productivity Commission. 2005. **Australia’s Health Workforce, Research Report**. Canberra: Commonwealth of Australia.

38. Hutton DW, Tan D, So SK, and Brandeau ML. 2007. **Cost-effectiveness of screening and vaccinating Asian and Pacific Islander adults for hepatitis B**. *Ann Intern Med* **147**(7): 460-9.

39. Yang HI, Lu SN, Liaw YF, You SL, Sun CA, Wang LY, Hsiao CK, Chen PJ, Chen DS, and Chen CJ. 2002. **Hepatitis B e antigen and the risk of hepatocellular carcinoma**. *N Engl J Med* **347**(3): 168-74.

40. Yuen MF, Yuan HJ, Hui CK, Wong DK, Wong WM, Chan AO, Wong BC, and Lai CL. 2003. **A large population study of spontaneous HBeAg seroconversion and acute exacerbation of chronic hepatitis B infection: implications for antiviral therapy**. *Gut* **52**(3): 416-9.

41. Tsang PS, Trinh H, Garcia RT, Phan JT, Ha NB, Nguyen H, Nguyen K, Keeffe EB, and Nguyen MH. 2008. **Significant prevalence of histologic disease in patients with chronic hepatitis B and mildly elevated serum alanine aminotransferase levels**. *Clin Gastroenterol Hepatol* **6**(5): 569-74.

42. Wang CC, Lim LY, Deubner H, Tapia K, Lau AW, Manansala J, Krows M, Shuhart MC, and Kowdley KV. 2008. **Factors predictive of significant hepatic fibrosis in adults with chronic hepatitis B and normal serum ALT**. *J Clin Gastroenterol* **42**(7): 820-6.

43. 2003. **EASL International Consensus Conference on Hepatitis B. 13-14 September, 2002: Geneva, Switzerland. Consensus statement (short version)**. *J Hepatol* **38**(4): 533-40.

44. Chang TT, Gish RG, de Man R, Gadano A, Sollano J, Chao YC, Lok AS, Han KH, Goodman Z, Zhu J, Cross A, DeHertogh D, Wilber R, Colonno R, and Apelian D. 2006. **A comparison of entecavir and lamivudine for HBeAg-positive chronic hepatitis B**. *N Engl J Med* **354**(10): 1001-10.

45. Liaw YF, Sung JJ, Chow WC, Farrell G, Lee CZ, Yuen H, Tanwandee T, Tao QM, Shue K, Keene ON, Dixon JS, Gray DF, and Sabbat J. 2004. **Lamivudine for patients with chronic hepatitis B and advanced liver disease**. *N Engl J Med* **351**(15): 1521-31.

46. Lavanchy D. 2004. **Hepatitis B virus epidemiology, disease burden, treatment, and current and emerging prevention and control measures**. *J Viral Hepat* **11**(2): 97-107.

47. Villeneuve JP, Condreay LD, Willems B, Pomier-Layrargues G, Fenyves D, Bilodeau M, Leduc R, Peltekian K, Wong F, Margulies M, and Heathcote EJ. 2000. **Lamivudine treatment for decompensated cirrhosis resulting from chronic hepatitis B**. *Hepatology* **31**(1): 207-10.

48. Tracey E, Chen S, Baker D, Bishop J, and Jelfs P. 2006. **Cancer in New South Wales: Incidence and Mortality 2004.** Sydney: Cancer Institute NSW.

49. Commonwealth Department of Health and Ageing. 2007. **Medicare Benefits Schedule Book**. Department of Health and Ageing. Canberra: Commonwealth of Australia.

50. Commonwealth Department of Health and Ageing. 2007. **National Hospital Cost Data Collection. Cost Report Round 10 (2005-6) AR-DRG v5.0.** C. D. o. H. a. Ageing. Canberra, ACT: Commonwealth Department of Health and Ageing.

51. Commonwealth Department of Health and Ageing. 2007. **Schedule of Pharmaceutical Benefits for approved pharmacists and medical practitioners**. Department of Health and Ageing. Canberra: Commonwealth of Australia.
