# Supplementary material for: Using a population-based approach to prevent hepatocellular cancer in New South Wales, Australia: effects on health services utilisation
Source: BMC Health Serv Res. 2010 Jul 21;10:215. doi: 10.1186/1472-6963-10-215 (PMC2918596; doi:10.1186/1472-6963-10-215)
Supplement: Additional file 2 — Table S1: Epidemiological transition probabilities for people with chronic hepatitis B managed under current practice and under the modelled HCC prevention program. CHB progressions states modelled using 6 discrete states; transition probabilities presented, together with data sources. [file 1472-6963-10-215-S2.DOCX]

Table S1 Epidemiological transition probabilities for people with chronic hepatitis B managed under current practice and the modelled HCC prevention program

| **Probability** | **Annual probability**  **or relative risk** | **Data source**  **(reference number)** |
| --- | --- | --- |
| *CHB patients* |  |  |
| Autoimmune cure; current practice | 1.5% | [43] |
| Autoimmune cure; prevention program | 2.0% | [44] |
| Develop cirrhosis; current practice | 2.1% | [17] |
| RR of cirrhosis with prevention program | 0.1 | Expert opinion |
| Develop HCC; current practice | 0.2% | [17] |
| RR of HCC with prevention program | 0.5 | [45] |
| *Cirrhosis patients* |  |  |
| Autoimmune cure; current practice | 1.5% | [43] |
| Autoimmune cure; prevention program | 2.0% | [44] |
| Develop liver failure; current practice | 5.6% | [46] |
| RR of liver failure with prevention program | 0.1 | Expert opinion |
| Develop HCC; current practice | 4.5% | [43] |
| RR of HCC with prevention program | 0.5 | [45] |
| *Patients with liver failure* |  |  |
| CHB-related death; current practice | 19.4% | [47] |
| RR of CHB-related death with prevention program | 1.0 | Expert opinion |
| *Patients with HCC* |  |  |
| CHB-related death; current practice | 37% | [48] |
| RR of CHB related death with prevention or surveillance program | 0.6 | [9] |

**Abbreviations:** CHB: chronic hepatitis B; HCC hepatocellular cancer; RR: relative risk
