# Supplementary material for: Using a population-based approach to prevent hepatocellular cancer in New South Wales, Australia: effects on health services utilisation
Source: BMC Health Serv Res. 2010 Jul 21;10:215. doi: 10.1186/1472-6963-10-215 (PMC2918596; doi:10.1186/1472-6963-10-215)
Supplement: Additional file 3 — Table S2: Cost of resources used up for routine hepatitis care, CHB surveillance and HCC prevention. Costs calculated for all tests and services utilised (except palliative care)-unit costs calculated; expert opinion used where costing not available (cost of chemoembolisation and viral load assay). [file 1472-6963-10-215-S3.DOCX]

Table S2 Cost of resources used up for routine hepatitis care, CHB surveillance and HCC prevention

| **Element** | **Schedule**  *Per year* | **Unit Cost**  *AU$* | **Source** |
| --- | --- | --- | --- |
| ***Routine care*** |  |  |  |
| GP consultation (standard) | 2 | 32.10 | MBS [49] |
| Two tests: HBsAg & HBeAg | 2 | 29.45 | MBS [49] |
| Hepatitis B DNA (HBV DNA) | 1 | 120.00 | Expert opinion |
| Liver function test (ALT) | 2 | 9.75 | MBS [49] |
| ***HCC surveillance*** |  |  |  |
| GP consultation (standard) | 2 | $32.10 | MBS [49] |
| Two tests: HBsAg & HBeAg | 2 | 29.45 | MBS [49] |
| Hepatitis B DNA (HBV DNA) | 1 | 120.00 | Expert Opinion |
| Liver function test (ALT) | 2 | 9.75 | MBS [49] |
| Alpha fetoprotein (AFP) test | 2 | 24.75 | MBS [49] |
| Ultrasound | 2 | 111.30 | MBS [49] |
| ***CHB treatment*** |  |  |  |
| Specialist consultation | 6 (interferon) or 4 (entecavir) | 56.74 | MBS [49] |
| Chemoembolisation (HCC not eligible for resection) |  | 314.40 | Expert Opinion |
| Radio frequency ablation (HCC not eligible for resection) |  | 722.05 | MBS [49] |
| Liver resection (all costs) |  | 27,196.00 | NHCDC  NHCDC [50] |
| Interferon | 180 mcg/week | 17,552.99 | PBS [51] |
| Entecavir | 0.5mg / day | 4,611.60 | PBS [51] |
| **Initial screening** |  |  |  |
| GP consultation (long) | 1 – for those HBsAg positive | 60.95 | MBS [49] |
| GP consultation (standard) | 1 – for those HBsAg negative | 32.10 | MBS [49] |
| Three CHB tests: HBsAg, HBeAg + one other | 1 | 40.80 | MBS [49] |
| Hepatitis B DNA (HBV DNA) | 1 | 120.00 | Expert Opinion |
| Liver function test (ALT) | 1 | 9.75 | MBS [49] |
| Specialist consultation (initial) | 1 for high risk patients | 113.86 | MBS [49] |
| Alpha fetoprotein (AFP) test | 1 for high risk patients | 24.75 | MBS [49] |
| Ultrasound procedure | 1 for high risk patients | 111.30 | MBS [49] |
| Biopsy (all costs) | 1 for high risk patients | 407.50 | MBS [49] |

**Abbreviations:** MBS: Medical Benefits Schedule; PBS: Pharmaceutical Benefits Schedule; NHCDC: National Hospital Cost Data Collection
