# Supplementary material for: Using a population-based approach to prevent hepatocellular cancer in New South Wales, Australia: effects on health services utilisation
Source: BMC Health Serv Res. 2010 Jul 21;10:215. doi: 10.1186/1472-6963-10-215 (PMC2918596; doi:10.1186/1472-6963-10-215)
Supplement: Additional file 4 — Table S3: Utility weights used in the economic model. Lists age-specific weightings used in the model and their derivation. [file 1472-6963-10-215-S4.DOCX]

Table S3: Utility weights used in the economic model

| **Age band** | **Cleared CHB** | **CHB only** | **Cirrhosis** | **HCC** | **Liver failure** |
| --- | --- | --- | --- | --- | --- |
| **35-44** | 0.91 | 0.87 | 0.84 | 0.41 | 0.46 |
| **45-54** | 0.85 | 0.81 | 0.78 | 0.35 | 0.40 |
| **55-64** | 0.80 | 0.76 | 0.73 | 0.30 | 0.35 |
| **65-74** | 0.78 | 0.74 | 0.71 | 0.28 | 0.33 |
| **75+** | 0.73 | 0.69 | 0.66 | 0.23 | 0.28 |
