# Supplementary material for: Using a population-based approach to prevent hepatocellular cancer in New South Wales, Australia: effects on health services utilisation
Source: BMC Health Serv Res. 2010 Jul 21;10:215. doi: 10.1186/1472-6963-10-215 (PMC2918596; doi:10.1186/1472-6963-10-215)
Supplement: Additional file 5 — Table S4: Sensitivity analysis on the estimated effects of the program on disease progression and number of medical appointments in year 4 of the program*. Sensitivity analysis of assuming different rates of reduction of cases progressing from CHB to cirrhosis, cirrhosis to liver failure and liver failure to death. [file 1472-6963-10-215-S5.DOCX]

Table S4: Sensitivity analysis on the estimated effects of the program on disease progression and number of medical appointments in year 4 of the program*

| **Treatment group** | **Incremental annual number of appointments due to disease surveillance and/ or treatment** | | |
| --- | --- | --- | --- |
|  | **GP** (averaged per GP) | **Specialist** (averaged per specialist) | **Liver ultrasound** |
| **Base case assumptions:**   - *90% risk reduction CHB to cirrhosis* - *90% risk reduction cirrhosis to liver failure* - *0% risk reduction liver failure to death* | **1,734**  *(5.0)* | **759**  *(25.3)* | **419** |
| **CHB to cirrhosis:** | | | |
| Assuming 70% risk reduction | **1,734**  *(5.0)* | **761**  *(25.4)* | **419** |
| Assuming 50% risk reduction | **1,734**  *(5.0)* | **762**  *(25.4)* | **420** |
| **Cirrhosis to liver failure** | | | |
| Assuming 70% risk reduction | **1,734**  *(5.0)* | **760**  *(25.3)* | **419** |
| Assuming 50% risk reduction | **1,733**  *(5.0)* | **760**  *(25.3)* | **419** |
| **Liver failure to death** | | | |
| Assuming 10% risk reduction | **1,734**  *(5.0)* | **759**  *(25.3)* | **419** |
| Assuming 50% risk reduction | **1,734**  *(5.0)* | **759**  *(25.3)* | **419** |

*Starting Year 4, after all recruitment has been completed
